# The Effect of High‐Intensity Interval Training and Mixed Probiotic Supplementation on SMOC‐1 Gene Expression, Insulin Resistance, and Blood Glucose in Male Rats With Induced Diabetes

**DOI:** 10.1155/jdr/5518066

**Published:** 2025-10-28

**Authors:** Dorsa Ghazvineh, Marjan Dodangeh, Sahar Razmjou, Alireza Rahimi, Mitra Azizi Masouleh

**Affiliations:** ^1^ Department of Exercise Physiology, Ka.C., Islamic Azad University, Karaj, Iran, azad.ac.ir; ^2^ Faculty of Health Sciences, School of Human Kinetics, University of Ottawa, Ottawa, Ontario, Canada, uottawa.ca

**Keywords:** diabetes, GM, HIIT, IR, mixed probiotics, SMOC-1

## Abstract

**Background and Aims:**

In this study, we investigated whether high‐intensity interval training (HIIT) and mixed probiotic consumption, either autonomously or synergistically, could regulate the expression of calcium‐binding protein‐1 (SMOC‐1), insulin resistance (IR), and blood glucose (BG) in male rats with induced diabetes.

**Methods:**

Thirty healthy male Wistar rats, aged about 8 weeks, were randomly divided into five groups of six rats each, including control group (C, 1G), diabetic control group (CD, 2G), probiotic supplement group (Pro, 3G), HIIT group (Ex, 4G), and HIIT and probiotic supplement group (Pro + Ex, 5G). Each strain of the mixed probiotic supplement, containing *Lactobacillus rhamnosus* GG (PTCC1637), *Lactobacillus reuteri*, and *Lactococcus casei* enriched with L‐cysteine HCl, was administered at a concentration of 10^10^ colony‐forming units (CFU) per milliliter to Groups 3G and 5G. Groups 4G and 5G underwent HIIT to evaluate the effect of supplementation and HIIT on SMOC‐1, IR, and BG.

**Results:**

Mixed probiotics and HIIT did not affect SMOC‐1 expression in liver muscle (*η* ^2^ = 0.00, *p* = 0.965, *F*_(1, 15)_ = 0.002); however, they synergistically lowered IR (*η* ^2^ = 0.23, *p* = 0.048, *F*_(1, 15)_ = 4.65) and BG (*η* ^2^ = 0.32, *p* = 0.013, *F*_(1, 15)_ = 7.79).

**Conclusion:**

We found no significant effect of mixed probiotic supplementation or its combination with HIIT on SMOC‐1. Notably, the HIIT and mixed probiotics reduced IR and BG. Future studies can help assess the accurate synergistic effects of HIIT and mixed probiotics.

## 1. Introduction

The liver has an imperative role in glucose storage as glycogen and glucose uptake through the gluconeogenic pathway after a meal. Additionally, this organ can package excess fat for secretion and storage in other tissues, such as adipose tissue, through lipid oxidation. It is also responsible for processing amino acids to produce energy and wiping out nitrogen waste resulting from protein breakdown through urea metabolism [1]. Consequently, the liver plays a key role in managing diseases associated with diabetes and obesity [2, 3].

Hepatokines are proteins secreted by the liver that play a role in regulating metabolism throughout the body. Montgomery et al. [4] identified secreted calcium‐binding protein‐1 (SMOC‐1) as a glucose‐responsive hepatokine that is crucial for glucose homeostasis in mice.

The SMOC‐1 gene is primarily found in basement membranes, where it binds with other proteins to regulate molecules like growth factors (GFs) that can either inhibit or promote mitosis and influence cellular differentiation. In individuals with diabetes or excessive weight, SMOC‐1 levels are usually reduced. However, lifestyle factors such as diet and physical activity (PA) can affect proper expression [5]. Initially, SMOC‐1 expression reduces the phosphorylation of cAMP response element‐binding protein Serine 133 (CREB Ser133). This reduction subsequently lowers the level of the enzyme phosphoenolpyruvate carboxykinase‐1 (Pck1) and catalyzes the hydrolysis of glucose‐6‐phosphate (G6pc). These events highlight the importance of SMOC‐1 in reducing insulin resistance (IR) and improving glucose tolerance [4], potentially through enhanced insulin‐regulated glucose transporter (GLUT4) activity. Additionally, since SMOC‐1 levels are decreased and IR increased in diabetic individuals, particularly those with higher body weight (BW), these levels can be balanced through fat loss and weight management. Therefore, it can be concluded that regular exercise and a balanced diet can favorably adjust SMOC‐1 and IR. Studies have shown that interval training, such as high‐intensity interval training (HIIT), significantly impacts weight management and helps prevent noncontagious diseases [6, 7].

Besides exercise training, dietary supplements have gained considerable attention among individuals to improve their well‐being. However, proper supplemental selection must be supervised by professionals. In this context, probiotics have recently been widely used in the supplement industry through different accessible supplements to help individuals address their physical and even mental disorders [8]. It is worth mentioning that several factors can affect the effectiveness of supplements, including PA, dietary patterns, and the presupplement cleansing period.

In this study, we aimed to decipher the effect of HIIT and combined probiotic consumption on the gene expression of SMOC‐1 hepatokines, blood glucose (BG), and IR in male rats with induced diabetes. While similar studies have investigated the impact of intense interval training or mixed probiotic consumption on hepatic cytokine expression in diabetic rats, this research adds value by combining both interventions. Additionally, SMOC‐1 is a promising therapeutic target; its proper expression could potentially offer diabetic patients an alternative medical intervention. The results of this study could help the medical community by providing insights that could reduce the financial burden and mitigate the side effects associated with chemical treatment for diabetes. Uniquely, this study focuses on a combined probiotic supplement, whereas previous studies have used only one type of probiotic strain or combined them with other aerobic exercises.

## 2. Method

### 2.1. Animal

Thirty healthy male Wistar rats, approximately 8 weeks old, were purchased from the Pasteur Institute′s Animal Breeding and Reproduction Center in Tehran, Iran. After their arrival at the Center for Laboratory Animal Sciences, the rats were given 2 weeks to get familiar with the new environment. They were housed in standard cages (42 × 26.5 × 15 cm) under a 12‐h light/dark cycle, with a temperature maintained at around 20°C (±3°C) and humidity levels between 10% and 50%. The rats were fed daily with standard laboratory food, receiving 10 g of food for every 100 g of their BW, and had free access to drinking water. This study was performed according to the Animal Research Reporting of In Vivo Experiments (ARRIVE) for animal care and animal experimentation. The study protocol was approved by the Ethical Committee of the Islamic Azad University of Alborz, Iran (Ethical Code: IR.IAU.K.REC.1402.134). Furthermore, the rats were randomly divided into five groups of six rats each:

• Control group (C, 1G): In this group, healthy rats did not exercise but were placed motionless on a stationary treadmill during the exercise sessions to experience the same environment as the other groups.
• Diabetic control group (CD, 2G): Similar to Group C, diabetic rats in this group did not exercise and were treated the same way, spending time on the stationary treadmill without PA.
• Probiotic supplement group (Pro, 3G): These diabetic rats did not exercise but were given a mixed probiotic supplement over 8 weeks to evaluate the impact of probiotics on their health.
• HIIT group (Ex, 4G): These diabetic rats took part in an 8‐week HIIT program, designed to appraise the effects of intense PA.
• HIIT and probiotic supplement group (Pro + Ex, 5G): Diabetic rats that received the probiotic supplement and participated in the 8‐week HIIT program concurrently.

### 2.2. Inclusion and Exclusion Criteria

Rats with any pre‐existing metabolic disorders, infections, or injuries were excluded from the study. Additionally, they were excluded from the study if they suffered from severe illness or weight loss of more than 20% of BW.

### 2.3. Type 2 Diabetes Mellitus (T2DM)

While the appropriate dose of streptozotocin (STZ) varies based on factors such as BW, age, and strain, a general range of 42–65 mg/kg is typically administered either intraperitoneally (IP) or intravenously (IV). Accordingly, all rat groups except for those in the 1G group (healthy control) were injected with STZ using a 0.5‐inch 25‐gauge needle on a tuberculin (1.0 mL) syringe with STZ. To avoid cytotoxicity and excessive insulin secretion into the bloodstream, the animals were given 10% sucrose water immediately after the injections. BG levels were then tested 48 h postinjection, after the STZ injection. If the rats were hyperglycemic at that time, they were returned to standard drinking water [9].

### 2.4. Probiotics

To prepare the mixed probiotic supplement, we cultured strains of *Lactobacillus rhamnosus* GG (PTCC1637), *Lactobacillus reuteri*, and *Lactococcus casei* in MRS medium (Biogene, Tehran, Iran), which was enriched with L‐cysteine HCl. The cultures were then incubated at the Microbial Sciences Research Center for 24 h at 37°C. Groups 3G and 5G received a bacterial suspension containing 10^10^ colony‐forming units (CFU) per milliliter of each strain to evaluate the effect of probiotic consumption. This suspension was administered via gavage for 8 weeks, 5 days a week (Table 1).

**Table 1:** Gut microbiota′s function.

**Probiotic**	**Property**
*Lactobacillus rhamnosus* GG (PTCC1637)	Reduce dysbiosis, reduce insulin resistance, and reduce FBS and HbA1c, gut barrier function, anti‐inflammation, lipid metabolism

*Lactobacillus reuteri*	Influence the production of hormones such as *GLP-1*, bone health, insulin sensitivity, anti‐inflammation, and GM balance

*Lactococcus casei*	Regulate metabolic processes, cure digestive disorders such as diarrhea, constipation, and IBS, and decrease proinflammatory cytokines

Abbreviations: FBS, fasting blood glucose; GLP‐1, glucagon‐like peptide‐1; GM, gut microbiota; HbA1c, hemoglobin A1c or glycated hemoglobin; IBS, irritable bowel syndrome.

### 2.5. Protocol HIIT Exercise

This protocol was conducted over 8 weeks, 5 days a week. The initial 2 weeks were allocated to familiarization, while the remaining 6 weeks involved performing the exercise protocol in the mornings, followed by gavage of the mixed probiotic supplement to the rats shown in Table 2 [10]. Maximum oxygen consumption (VO_2max_) was measured after rats ran on a treadmill at a speed of 6 m/min on a zero‐degree incline for 5 min of warm‐up. Subsequently, the treadmill speed was increased by 3 m/min every 3 min until the animal reached exhaustion [11].

**Table 2:** Exercise training protocol.

**Training components**	**Warm-up**	**HIIT**	**Active rest**	**Cool down**
Time of training	5 min	2 min	1 min	5 min
First week (m/min)	5	24–27	10–11	5
Second week (m/min)	5	32–36	11–12	5
Third week (m/min)	5	36–40	13–14	5
Fourth week (m/min)	5	41–45	14–15	5
Fifth week (m/min)	5	46–50	15–16	5
Sixth week (m/min)	5	51–55	16–17	5
Exercise intensity	80%^a^ with zero‐degree incline on the treadmill			

Abbreviations: HIIT, high‐intensity interval training; m, meter; min, minute.

^a^VO_2max_ 80%.

### 2.6. Euthanasia and Organ Extraction

Twenty‐four hours after the last session of the training, the rats were anesthetized with intraperitoneal injection of a ketamine (90 mg/kg) and xylazine (10 mg/kg) mixture. After complete anesthesia, the liver muscle was immediately extracted, rinsed with physiological saline, and then snap‐frozen in liquid nitrogen and stored at −86°C until further analysis. During tissue sample preparation, they were first removed from the freezer and allowed to reach room temperature, then weighed, and 56–266 mg of each sample was placed into a microtube.

### 2.7. Histological Analysis

Initially, 20–40 mg of the tissue sample were isolated and cleaned using a sterile scalpel before being placed into a sterile petri dish. The sample was then finely chopped until a slurry was obtained, which was transferred into a sterile microtube until the solution flowed freely. Then, 750 *μ*L of the lysis buffer from the Parstous Company Extraction Kit was added. The homogenization process was carried out using a homogenizer under ice conditions until the lysed tissue was completely homogenized, and it was incubated at room temperature for 5 min. Subsequently, 150 *μ*L of chloroform was added and mixed thoroughly, and the mixture was incubated for 3 min at room temperature. The sample was then centrifuged at 4°C for 12 min at 13,000 rpm. After centrifugation, 400 *μ*L of the supernatant was transferred into a new microtube, and 400 *μ*L of 70% ethanol was added thoroughly. This solution was placed in a new column and centrifuged at 13,000 rpm for 1 min. The resulting liquid was poured out, and after that, 700 *μ*L of wash buffer was added to the column, followed by another centrifugation at 13,000 rpm for 1 min. This wash step was repeated once more to dry the column without adding any additional solutions. Ultimately, 50 *μ*L of DEPC‐treated water was added, and the contents were transferred to a new microtube and centrifuged at 13,000 rpm for 1 min to collect the final product [12].

### 2.8. Gene Expression

To prepare the Master Mix for PCR reactions, we followed the protocol provided by the Pasteur Institute of Iran, a medical research center. Total RNA was isolated using a guanidine/phenol solution (QIAzol, QIAGEN, United States). RNA quality and quantity were appraised by NanoDrop 2000 (Thermo Scientific). Based on the manufacturer′s protocol, 1 *μ*g of total RNA was taken for the first‐strand complementary DNA (cDNA) synthesis reaction (HyperScript RT‐PCR, GeneAll). We conducted the real‐time reaction in PCR tubes with a final volume of 20 *μ*L, using a CFX384 Bio‐Rad thermal cycler (Bio‐Rad) for amplification. Each sample′s cDNA (1 *μ*L) was amplified using SensiMiX (Bioline) SYBR Green PCR, consisting of 10 *μ*L of Master Mix (2×) (Applied Biosystems, Amplicon, Catalog Number 4309155) with 7500 Fast Sequence detector (Applied Biosystems, Foster City, CA, United States), 1 *μ*L of forward primer, 1 *μ*L of reverse primer, 1 *μ*L of cDNA, and finally 7 *μ*L of DEPC‐treated water adjusted according to the type of genes and amplification temperature protocol. The housekeeping gene GAPDH was measured concurrently as an internal control. The thermal profile employed for the qRT‐PCR had three stages: the initial stage, 95°C for 3 min (1 cycle); the amplification stage with cycles at 95°C, 57°C, and 72°C for 30 s each (40 cycles); and a final stage at 95°C for 15 s, followed by 60°C for 1 h (1 cycle). The fold change for each gene was determined after normalization to GAPDH using the 2^−*ΔΔ*CT^ method [13]. *Δ*CT = CT_target_ − Ct_reference_; *Δ* *Δ*Ct = *Δ*CT_test sample_ − *Δ*CT_control sample_, relative expression: 2^−*ΔΔ*Ct^. Results are presented as the mean ± standard deviation (SD). The primer sequences are also shown in Table S1.

### 2.9. Statistical Methods

IBM SPSS Version 26 software (IBM Corporation, Armonk, NY) was used to analyze the data. Descriptive statistics, including means and SDs, were calculated to summarize the data. The Shapiro–Wilk test was performed to assess if the data were normally distributed. For inferential statistics and hypothesis testing, a two‐way analysis of variance (ANOVA) was conducted, with a significance level set at *α* < 0.05. Additionally, Levene′s test was employed to evaluate the equality of variances.

## 3. Results

Data from 25 remaining rats showed that their BW had modifications compared to the baseline; 250 ± 20 had modifications. Group 4G experienced the most significant reduction, dropping to 160 ± 16.2 g. In contrast, the other groups saw a slight increase in weight: 1G: 260 ± 21 g, 2G: 269.4 ± 6.6 g, 3G: 256.2 ± 32.4 g, and 5G: 275 ± 16.03 g, as shown in Table S2.

In addition, SMOC‐1 expression was highest in Group 2G with 27.35 ± 1.04. Group 4G ranked second in gene expression with 27.13 ± 1.88, while Groups 3G and 5G showed almost similar levels of expression with 25.78 ± 0.70 and 25.62 ± 1.80, respectively. This shows a notable disparity between healthy male rats in Group 1G (25.51 ± 1.05) and diabetic rats in Group 2G (27.35 ± 1.04) (Table S3). Furthermore, the Shapiro–Wilk test showed that all groups had *p* > 0.05, suggesting no significant differences.

Based on the two‐way ANOVA for SMOC‐1, HIIT had a significant impact on male diabetic rats (*η* ^2^ = 0.25, *p* = 0.039, *F*_(1, 15)_ = 5.09). This indicates the HIIT period led to a significant decrease in SMOC‐1 gene expression. In contrast, combined probiotic supplementation showed no differences in SMOC‐1 gene expression in male diabetic rats (*η* ^2^ = 0.00, *p* = 0.785, *F*_(1, 15)_ = 0.077), suggesting that probiotic supplementation did not have a beneficial effect on SMOC‐1 gene expression. Finally, the interaction effect of HIIT and mixed probiotic supplementation on SMOC‐1 gene expression in male diabetic rats was also not significant (*η* ^2^ = 0.00, *p* = 0.965, *F*_(1, 15)_ = 0.002), indicating that the combination of HIIT and probiotics did not result in substantial changes in SMOC‐1 gene expression compared to either intervention autonomously (Table S3).

Furthermore, the combination of HIIT and probiotics helped reduce IR by 22.42 ± 1.5; in addition, the levels of reduction for HIIT and probiotics ranked second with 26.68 ± 1.16 and 26.27 ± 2.2, respectively. Group 2G saw the highest IR 27.18 ± 1.41, and Group 1G had the lowest levels 23.75 ± 0.98 (Table S3).

According to the two‐way ANOVA for IR, probiotics had a considerable impact on male diabetic rats (*η* ^2^ = 0.42, *p* = 0.005, *F*_(1, 15)_ = 11.08). This shows that the probiotic improved insulin sensitivity. Then, HIIT showed less impact on IR in male diabetic rats (*η* ^2^ = 0.34, *p* = 0.013, *F*_(1, 15)_ = 7.86). Finally, the interaction effect of HIIT and mixed probiotic supplementation on IR depicted the lowest influence (*η* ^2^ = 0.23, *p* = 0.048, *F*_(1, 15)_ = 4.65) (Table S3).

Based on the two‐way ANOVA for BG, probiotics had a considerable impact on the BG in male diabetic rats (*η* ^2^ = 0.39, *p* = 0.005, *F*_(1, 15)_ = 10.53). This shows that the probiotic improved BG. Then, HIIT showed no impact on BG in male diabetic rats (*η* ^2^ = 0.16, *p* = 0.099, *F*_(1, 15)_ = 3.07). Finally, the interaction effect of HIIT and mixed probiotic supplementation on BG depicted no influence (*η* ^2^ = 0.32, *p* = 0.013, *F*_(1, 15)_ = 7.79) (Table S4).

## 4. Discussion

Consumption of mixed probiotics did not significantly impact SMOC‐1 expression in liver muscle. However, HIIT caused a sharp drop in the expression of SMOC‐1. Additionally, the interaction of probiotic supplementation and HIIT did not result in any changes to this gene. Besides, the HIIT and mixed probiotics reduced IR either autonomously or synergistically. However, HIIT could reduce the levels of BG, but probiotics could not affect it significantly.

Previous research highlighted the clear and snap impact of SMOC‐1 on BG in insulin‐resistant and diabetic rats. Montgomery et al. [4] surprisingly found that prescribing SMOC‐1 to insulin‐resistant and obese mice, after 8 weeks on a high‐fat diet, improved glucose tolerance. Several studies assessed the gene expression function of probiotics. For example, Palacios et al. showed the effects of a new probiotic supplement on metabolic biomarkers in adults with prediabetes and those recently diagnosed with T2DM. This supplement contained eight strains from the genera *Bifidobacterium*, *Lactobacillus*, *Saccharomyces*, and *Streptococcus* intended to promote glucose metabolism in individuals with prediabetes. They concluded that changes in gut microbiota (GM) induced by this multistrain probiotic reduced inflammatory and metabolic markers and improved BG [14]. Besides, Shima et al. showed that different probiotic strains can differentially affect gene expression in the colon and the small intestine. For instance, *L. casei* Shirota enhances lipid metabolism and immune function genes more effectively than *Bifidobacterium breve* Yakult [15]. In 2017, a study by Tonucci et al. [16] showed that probiotics tend to influence lipid metabolism, increase the number of glucose transporters in tissues such as GLUT4, and inhibit liver cholesterol synthesis through short‐chain fatty acids (SCFAs), leading to reduced oxidative stress and IR. Bayat et al. claimed the effects of 8 weeks of HIIT combined with a specific strain of *Lactobacillus* increased insulin sensitivity and adiponectin levels to some extent in a mouse model. Ultimately, the combination of exercise and probiotic supplementation may effectively impact both metabolic health and cognitive function by improving insulin sensitivity and adiponectin levels [17]. Bayat et al. in another study claimed that probiotic *Lactobacillus* combined with HIIT effectively decreases BW, visceral fat (VF), low‐density lipoprotein (LDL), glucose, and insulin and increases adiponectin levels. Notably, exercise is more effective in reducing fat and using probiotics for IR [18]. Hsieh et al. [19] concluded that *L. reuteri* strains ADR‐3 and ADR‐1 have an effective impact on T2DM patients and may change intestinal flora. Ogawa et al. [20] found that consuming probiotic LG2055 reduced postprandial and fasting serum nonesterified fatty acid (NEFA) levels, which reduces the risk of obesity and T2DM. Abasi et al. reported that BG levels in the diabetic group were considerably increased compared to the control group (*p* < 0.001); however, the diabetic group treated with *Bifidobacterium lactis* and *L. casei* significantly improved compared to the control group (*p* < 0.001) [21]. Al‐Salami et al. [22] claimed that diabetic rats, which were treated with probiotics, increased gliclazide bioavailability and lowered BG levels by insulin‐independent mechanisms, suggesting that the administration of probiotics may be beneficial as adjunct therapy in the treatment of diabetes. Pegah et al. [23] showed that probiotics and resveratrol, by reducing oxidative stress and increasing GLP‐1 levels, can control T2DM. Zhang et al. concluded that the effects of both exercise and probiotic supplementation on reducing plasma glucose may be due to their combined influence on the number of GLUT4 transporters and increasing their expression. This synergistic effect highlights the potential for improved glucose regulation through the interplay of these two interventions [24]. Jelleyman et al. [25] also showed that HIIT has promising effects on IR and glucose regulation. Besides, compared to the control group, HIIT led to a decrease in BW and hemoglobin A1c (HbA1c) [25]. Contrary to our findings, Cassidy et al. [26] pointed out that HIIT improved glycemic control but has a restricted effect on cardiovascular autonomic regulation in patients with T2DM. D′Haese et al. [27] claimed that HIIT and moderate‐intensity training (MIT) positively affected pathological cardiac remodeling and diastolic dysfunction in T2DM, by optimizing mitochondrial capacity and lowering fibrosis. Sun et al. [28] showed that combining a low‐carbohydrate (LC) diet with exercise training may positively affect gut physiology.

### 4.1. Mechanism

Probiotic function can be affected by various factors including age [29], sex [30], medical history [31], level of PA [32], dietary choices [33], sleep duration [34], length of intervention [35], medications, supplements [36, 37], BW [38], and body mass index (BMI) [39]. For instance, research has shown that different exercise intensities can raise specific microbial communities, including butyrate producers and health‐promoting bacteria [40].

According to our results, physiological, biochemical, and molecular processes cause changes in a specific condition for BG and IR;

#### 4.1.1. Improve Insulin Signaling

Exercise can increase the activity of insulin receptors such as IRS‐1 and IRS‐2, enhancing insulin signaling in cells [41]. Probiotics may enhance insulin sensitivity by improving GM composition, particularly reducing inflammation [42–44]. Besides, they have insulin‐sensitizing effects following their potency to release SCFAs, which assist in adipocyte insulin signaling, block fat accumulation in adipose tissue, and reduce inflammation [45–47].

#### 4.1.2. Increase BG Uptake by Muscles

Onset exercise, especially HIIT, increases the expression of GLUT4, which enhances glucose uptake into muscle cells and lowers BG levels. Firstly, exercise activates Protein Kinase B (AKT), which plays a key role in increasing the expression of GLUT4 transporters and insulin signaling, leading to improved BG uptake. It also regulates microRNA 223 (miR‐223) and reduces TLR4 expression, contributing to the decrease in IR [48]. However, the duration of exercise potentially affects BG reduction; for instance, moderate‐duration HIIT interventions of more than 8 weeks were more effective in reducing BG, especially in individuals with impaired glucose [49]. However, if compensatory mechanisms such as increased glucose production in the liver are activated, this effect may become less noticeable [50].

#### 4.1.3. Reduce Systemic Inflammation and Improve Mitochondrial Function

Chronic inflammation, which is observed in T2DM, is a key factor in IR. Exercise and probiotic consumption can help reduce inflammation and oxidative stress, thereby improving the function of beta cells in the pancreas [51]. Besides, HIIT improves mitochondrial function, leading to more efficient energy utilization and a reduction in IR [52].

#### 4.1.4. Glucose Regulation in the Liver (Glyconeogenesis)

Exercise can increase hepatic glucose production to meet demands during PA, while this effect can diminish over time due to the body′s adaptation to the activity levels. Additionally, an increase in epinephrine during PA reduces the effect of BG reduction [53]. HIIT can increase plasma catecholamine levels such as noradrenaline and adrenaline, enhancing glucose production through regular strenuous activity, ultimately [54]. On the other hand, probiotics may influence this pathway by modulating GM and reducing hepatic glucose production [55], which can explain glucose reduction in the probiotic and combined groups.

In summation, BG regulation is more complex and may be affected by other factors, including improving mitochondrial function and increasing glucose uptake by muscles. However, BG levels may not be significantly reduced due to compensatory mechanisms such as hepatic glucose production. In contrast, probiotics likely improved both IR and BG levels by reducing inflammation and modulating GM. Hence, the combination of both supplement and exercise created synergistic effects, leading to improvement in both parameters.

#### 4.1.5. Duration of Exercise

One session of such exercise activities enhances insulin sensitivity and improves glucose tolerance for more than 24 h, but less than 72 h [56–59]. According to the present research, a longer duration of 3–6 months may be more effective. Thus, the length of exercise could be another significant factor, following intensity, in managing IR.

### 4.2. Regarding SMOC‐1 Changes

#### 4.2.1. Age of Subjects

Continuous vigorous exercise can pose risks and discomfort, particularly for those with T2DM, middle‐aged individuals, and those who have lower physical fitness and higher cardiovascular risk. Following these, SMOC‐1 levels decrease and exacerbate the situation [29, 60]. Another factor to consider is age; for example, older adults experience a significant decline in gut microbial density and diversity, which can reduce metabolic activity and physiological abnormalities. As a result, there tends to be a lower abundance of *Bifidobacteria* and a greater presence of Enterobacteriaceae, which may cause a decrease in the production of essential vitamins, such as vitamin B and enzymes [36].

#### 4.2.2. Increase Reactive Oxygen Species (ROS)

While probiotics inhibit the function of ROS [61], HIIT can enhance their activity [62]. ROS, such as cyclooxygenase, nitric oxide, and nitric oxide synthase, can increase nuclear factor kappa B (NF‐*κ*B) signaling associated with proinflammatory genes. This can activate insulin‐like growth factor‐1 (IGF‐1), which is linked to diseases such as T2DM, cardiovascular disease (CVD), and hypertension (HTN). Additionally, ROS can activate the CREB‐activated protein kinase pathway, which relates to gluconeogenesis. While probiotics can mitigate this process, intense exercises raise ROS levels irregularly.

#### 4.2.3. miR‐223

SMOC‐1 regulates miR‐223. miR‐223 is a short RNA molecule that regulates the expression levels of other genes through various mechanisms. Following vigorous exercise, miR‐223 may be overexpressed, and the downregulation of SMOC‐1 prevents the appropriate expression of GLUT4 [63, 64]. Hence, intensive activities may exacerbate the side effects of T2DM.

#### 4.2.4. Medication

Antibiotics have been demonstrated to make significant changes in the microbial environment of the body [36].

#### 4.2.5. Pathogen Microbiotas

Besides, stress levels and increased intestinal permeability induced by intense exercise may trigger the growth of pathogenic microbes like *Escherichia coli* and impede the reproduction of beneficial microbiotas such as *Lactobacillus* and *Bifidobacteria*, thereby inhibiting the proper function of probiotics [62, 65]. Hence, high‐intensity exercises performed more frequently can increase ROS and stress hormones such as cortisol, leading to excessive fat accumulation. When combined with an increase in *E. coli*, this can inhibit the function of helpful probiotics and GMs.

In summation, these factors interfere with the potential synergistic effect of HIIT and mixed probiotics. Thus, enriching individuals′ GMs, particularly older adults, with an appropriate dosage of probiotics can help maintain their overall well‐being. Furthermore, the total duration of the intervention can impact changes in glucose levels.

### 4.3. Strengths of the Study

All the rats were healthy at the beginning of the protocol, and they all became diabetic correctly at the next stage. They demonstrated good cooperation during this period and were kept in a similar situation. In addition, all devices used in the study were of high precision, and the measurements were supervised by a leader to ensure accuracy.

### 4.4. Limitations of the Study

There were some limitations in this research, including the following:

• Lack of precise control over the potential stress caused by the treadmill device and the mandatory exercise protocol is a concern.
• The study involved a small sample size of only 25 male Wistar rats, which were used to run and assess the considered protocols.

### 4.5. Recommendations for Other Researchers

• It would be beneficial to conduct a similar study with a larger sample size and control for factors that may influence the variables.
• Researchers should consider the interactive effects of mixed probiotic supplementation at different doses in combination with various intensities of HIIT.
• It is proposed to consider a proper interval between HIIT and probiotic intake to minimize potential interactions and enhance any synergistic effect.
• Future studies are recommended to include human subjects.
• Investigate the effect of mixed probiotic supplementation and HIIT on important indices involved in the hypoglycemic pathway, such as upstream or downstream factors of the SMOC‐1 signaling pathway.
• Additionally, it is recommended to evaluate the implemented protocols over a longer duration, exceeding 8 weeks, with a lower frequency during each week.
• Consider using other strains of probiotics that may be effective in diabetes management at different doses and quantities.
• It is also suggested to include a control group that receives a placebo treatment for comparison.

## 5. Conclusion

We found no significant effect of mixed probiotic supplementation or its combination with HIIT on SMOC‐1. Notably, the HIIT and mixed probiotics reduced IR and BG. Future studies are needed to assess the accurate synergistic effects of HIIT and mixed probiotics.

## Ethics Statement

The study protocol was approved by the Ethical Committee of the Islamic Azad University of Alborz, Iran (Ethical Code: IR.IAU.K.REC.1402.134).

## Conflicts of Interest

The authors declare no conflicts of interest.

## Author Contributions

All authors contributed to drafting the manuscript, reviewing the results, and approving the final version of the manuscript.

## Funding

No funding was received for this manuscript.

## Supporting information


**Supporting Information** Additional supporting information can be found online in the Supporting Information section. Table S1: The sequences of insulin receptor substrate (IRS1) used as the primer. Table S2: Body weight changes at the beginning of 8 weeks and the end of 8 weeks. Table S3: Levels of SMOC‐1 expression and IR as mean ± SD and ∗F(Sig.). Table S4: Levels of glucose as mean ± SD and ∗F(Sig.).

## Data Availability

All the data is available upon request.
