# Supplementary material for: The Effect of High‐Intensity Interval Training and Mixed Probiotic Supplementation on SMOC‐1 Gene Expression, Insulin Resistance, and Blood Glucose in Male Rats With Induced Diabetes
Source: J Diabetes Res. 2025 Oct 28;2025:5518066. doi: 10.1155/jdr/5518066 (PMC12585796; doi:10.1155/jdr/5518066)
Supplement: Supplementary file 1 — Supporting Information Additional supporting information can be found online in the Supporting Information section. Table S1: The sequences of insulin receptor substrate (IRS1) used as the primer. Table S2: Body weight changes at the beginning of 8 weeks and the end of 8 weeks. Table S3: Levels of SMOC‐1 expression and IR as mean ± SD and ∗F(Sig.). Table S4: Levels of glucose as mean ± SD and ∗F(Sig.). [file JDR-2025-5518066-s001.docx]

**Supplementary Table 1: The sequences of the insulin receptor substrate (IRS1) used as the primer**

| **Primer sequence** | **Genes** |
| --- | --- |
| Forward: 5-ATG TTC AGG TCC AGT CA-3  Reverse: 5-ACC AAG TGC TTA ATC CAT AA-3 | **SMOC-1** |
| Forward: 5-AGT GCC AGC CTC GTC TCA TA-3  Reverse: 5-TGA ACT TGC CGT GGG TAG AG-3 | **GAPDH** |

SMOC-1: secreted calcium-binding protein-1, GAPDH: glyceraldehyde 3-phosphate dehydrogenase

**Supplementary Table 2: Body Weight Changes at the beginning of 8 weeks and the end of 8 weeks**

| **At the end of 8 weeks(Mean±SD)** | **At the start of 8 8-week(Mean±SD)** | **Group** |
| --- | --- | --- |
| 260**±**21 | 250**±**20 | **C, 1G** |
| 6.6**±**269.4 |  | **CD, 2G** |
| 32.4**±**256.2 |  | **Pro, 3G** |
| 160**±**16.2 |  | **Ex, 4G** |
| 16.03**±**275.8 |  | **Pro + Ex, 5G** |

SD: Standard deviation, HIIT: High-intensity interval training, BW: Body weight, C, 1G: Control Group, CD, 2G: Diabetic Control Group, Pro, 3G: Probiotic Supplement Group, Ex, 4G: HIIT Group, Pro + Ex, 5G: HIIT and Probiotic Supplement Group

**Supplementary Table 3: Levels of SMOC-1 Expression and Insulin Resistance (IR) expressed as mean±SD, with significance indicated by *F(Sig.)**

| **Insulin Resistance (Mean±SD)** | **SMOC-1 (Mean±SD)** | **Group** |
| --- | --- | --- |
| 23.75±0.98 | 25.51±1.05 | **C, 1G** |
| 27.18±1.3 | 27.35±1.04 | **CD, 2G** |
| 26.27±1.2 | 25.78±0.70 | **Pro, 3G** |
| 26.68±1.1 | 27.13±1.88 | **Ex, 4G** |
| 22.42±1.5 | 25.62±1.8 | **Pro + Ex, 5G** |
| 7.86(.013) | 5.09(.039)^*^ | **Ex, 4G** |
| 11.08(.005) | .077(.785) | **Pro, 3G** |
| 4.65 (.048) | .002(.965) | **Pro + Ex, 5G** |

SD: Standard deviation, HIIT: High-intensity interval training. *F(Sig.)

**Supplementary Table 4: Levels Of Glucose as mean±SD, with statistical significance shown as *F(Sig.) between groups**

| **Control (Mean±SD)** | **HIIT (Mean±SD)** | **Group** |
| --- | --- | --- |
| 170±10 | 190±10 | **The group consumed a Probiotic** |
| 250±15 | 200±15 | **The group didn't consume the Probiotic** |
| 3.07(.099)^*^ | | **Ex, 4G** |
| 10.53(.005) | | **Pro, 3G** |
| 7.79(.013) | | **Pro + Ex, 5G** |

*F(Sig.)
